# Factors Associated with the Integration of Culturally and Linguistically Diverse Nurses into Healthcare Organisations: A Systematic Review of Quantitative Studies

**DOI:** 10.1155/2024/5887450

**Published:** 2024-06-24

**Authors:** R. Joensuu, K. Suleiman, M. Koskenranta, H. Kuivila, A. Oikarainen, J. Juntunen, Y. S. Goh, S. Y. Liaw, K. Mikkonen

**Affiliations:** ^1^ Research Unit of Health Sciences and Technology University of Oulu, Oulu, Finland; ^2^ Department of Nursing University of Otago, Christchurch, New Zealand; ^3^ Department of Healthcare and Social Services JAMK University of Applied Sciences, Jyvaskyla, Finland; ^4^ Health Care and Social Services Vaasa University of Applied Sciences, Vaasa, Finland; ^5^ Alice Lee Centre for Nursing Studies National University of Singapore, Queenstown, Singapore; ^6^ Medical Research Center Oulu Oulu University Hospital and University of Oulu, Oulu, Finland

## Abstract

**Background:**

Global nursing shortages have led to the recruitment of culturally and linguistically diverse nurses from various countries. However, nurses face integration challenges in their host countries.

**Objective:**

This systematic review aimed to find the most recent evidence of factors associated with integrational strategies and models to support the transition and adaptation of culturally and linguistically diverse nurses to the professional workforce in healthcare settings.

**Methods:**

This systematic review used the population, exposure, outcome framework to select studies according to JBI guidelines. Original peer-reviewed quantitative studies published between 2000 and 2021 were identified. Two researchers independently screened the studies based on eligibility criteria using title, abstract, and full text. The JBI critical appraisal tool assessed the methodology's quality for analytical cross-sectional studies. Data were extracted, tabulated, and analysed narratively. PRISMA checklist was used in reporting. *Data Sources*. CINAHL (Ebsco), PubMed, Medic, ERIC (ProQuest), and Scopus.

**Results:**

The systematic review encompassed 19 articles and identified multiple factors associated with successful integration. These factors were classified into the following six categories: sociodemographic characteristics, discrimination, social support, organisational support, workplace environment, and acculturation.

**Conclusions:**

Comprehensive cultural competency training for healthcare staff, including managers, enhances cultural proficiency in work environments. Clear guidelines addressing bias and discrimination create a supportive environment where culturally and linguistically diverse nurses feel valued and respected, facilitating their adaptation and integration. *Relevance to Clinical Practice*. Patient care quality can be improved by ensuring sustainable culturally and linguistically diverse nurses' integration into healthcare organisations. Cultural diversity is a unique opportunity to bring a diverse range and experience to clinical settings. The diversity can also help enhance the cultural competence of healthcare staff, allowing them to better understand and cater to the needs of culturally diverse patients. *Patient or Public Contribution*. Not required for study design.

## 1. Introduction

Globally, one in every eight nurses (13% = 3.7 million) were born or trained in a country other than the one in which they currently practise, and this mobility is expected to increase [1]. Recruitment of culturally and linguistically diverse (CALD) nurses can be considered a strategy to address nursing shortages and bring diverse backgrounds, experiences, and skills that can contribute to filling the gaps in the nursing workforce [1–4]. However, it is creating new challenges for healthcare organisations [5] and the host nurses who supervise the workplace entry of CALD nurses and assist them in integrating into the health workforce [6]. These challenges include, for example, learning a new language or technical terminology [7], differences in nursing practices and cultural values, discrimination and racism, or delays in recognition of competencies, all of which can lead to deskilling and frustration [8–10]. CALD nurses are often required to adapt and learn, while representatives of the majority population are generally not expected to change in multicultural work communities [4, 11, 12]. Moreover, working communities often have a normative requirement to adhere to established operating models whose contents may not be accustomed to negotiation. Hence, a good nurse with a foreign background has assimilated into the majority population as much as possible. In this case, support of the work community towards CALD nurses appears to be conditional; thus, if the newcomer CALD nurse is considered to be active and positive, the community may support them better [4].

Successful workplace integration is a continuous [13], complex, costly [14], and time-consuming process not only for the organisation but also for internationally trained nurses and host nurses [4, 15–18]. Integration is a two-way adaptation process or goal between the receiving society and the immigrant, as individuals and as a group, ensuring that both parties maintain their cultural identity [16, 19]. In integration, the immigrant commits and becomes accepted into the society of the destination country culturally, politically, and socioeconomically [19]. Factors in the workplace that promote the integration and permanence of CALD nurses are a practical work environment, cooperative work community, support from managers and colleagues, professional development opportunities [18, 20, 21], sociocultural support and training [20], and a desirable work-life balance [18]. Successfully integrated nurses can efficiently lead the work team in work communities, instruct colleagues, and participate in developing high-quality service [13, 17]. On the other hand, the lack of support for integration can cause weakened self-esteem, the feeling of being an outsider, shame, anxiety caused by a different culture, and isolation among immigrant nurses [11, 13].

The factors associated with integration into a different healthcare setting have been shown to impact CALD nurses' personal and professional outcomes significantly [13], patients and their families [22], and thus, the efficiency and profitability of the entire healthcare system [23]. Therefore, any strategies and models developed and implemented must adopt a multidimensional approach, considering factors associated with integrating CALD nurses. Considering these aspects, the integration process can be strengthened, leading to more positive outcomes [20, 24]. Poor workplace integration experience is intrinsically linked to high attrition rates of CALD nurses and concomitantly increasing healthcare costs while compounding existing nursing workforce shortages [25, 26].

There appears to be a lack of previous reviews of understanding the factors related to the integration strategies and models of CALD nurses and the potential for these factors to enhance outcomes during the transition of immigrant nurses into the professional workforce. The main objective of this review is to seek the latest evidence concerning the factors related to integrational strategies and models, thereby aiming to support the transition and adaptation of CALD nurses within healthcare environments. In addition, this research emphasises the importance of establishing the standards of healthcare organisations associated with the effective integration of CALD nurses.

## 2. Research Aim

This systematic review sought the most recent evidence on factors associated with integrational strategies and models to support CALD nurses' transition and adaptation to the professional workforce in healthcare settings.

The following research question is addressed: which factors are associated with integrational strategies and models developed to support the transition and adaptation of CALD nurses to the professional workforce in healthcare settings?

## 3. Methods

### 3.1. Search Strategy

This systematic review was conducted following the guidelines of the Joanna Briggs Institute (JBI) Manual for Evidence Synthesis—Systematic Review [27]. Systematic reviews aim to establish evidence by synthesising international research, and the results are used to inform practice, policy, evidence-based practice, and research. The advantages of conducting a systematic review are that the findings establish the international scope of practice, help build new practices by either affirming or putting to question current practice, and support clinical decision-making [28]. This method suited our phenomena since CALD nurse integration into healthcare is global, and findings may help improve integrational practices. After identifying the aim of the study and research question [29], participants, exposure of interest, and outcomes' (PEOs) protocol was used to formulate the review inclusion and exclusion criteria [28]. The review participants included CALD nurses from primary and specialised healthcare settings. Primary healthcare settings included general practice, primary care clinics, community health centres, emergency care, and residential older person care facilities. Specialised healthcare settings included hospitals, speciality clinics, rehabilitation centres, and mental health facilities. Students, patients, and healthcare professionals other than nurses were excluded from this study. The exposure of interest was studies that describe factors that predict or are related to integrating CALD nurses into healthcare organisations. The review searched for studies that reported outcomes of CALD nurses' models and strategies for integrating into nursing professional work in primary and specialised healthcare organisations, including orientation and education, team and atmosphere at work, competence evaluation and career development, management and leadership, mentorship, and retention. The review examined original, peer-reviewed, quantitative studies published from 2000 to 2021. The language limitation was set to Finnish and English. Grey literature was not included. Qualitative studies were excluded from this systematic review since they did not align with the research aim.

The search terms used included synonyms of the population, exposure, outcome (PEO) framework keywords relevant to this study as inclusion criteria [27]. The Oulu University library and researchers in the subject area were consulted to ensure that appropriate databases, search terms, and keywords were included to enhance the validity of the information retrieved for this review. The search was focused on the inclusion criteria and combined with Boolean operators AND, OR, and NOT (see Supplementary File 1).

Five databases were selected to retrieve original studies for the systematic review: PubMed, CINAHL (EBSCO), ERIC (ProQuest), Scopus, and Medic. PubMed stood out for its user-friendly interface and comprehensive content coverage, leading to its selection over Ovid Medline. In addition, Medic, a Finnish health sciences reference database managed by the Helsinki University Library, was considered. It encompasses medical, dental, nursing, and associated bioscience literature alongside selected publications from other pertinent fields. Hosting about 120,000 references, it sees an annual addition of roughly 3,000 new entries, focusing on material published in Finland, irrespective of language [30].

#### 3.1.1. Screening Process and Quality Assessment

**Figure 1:**
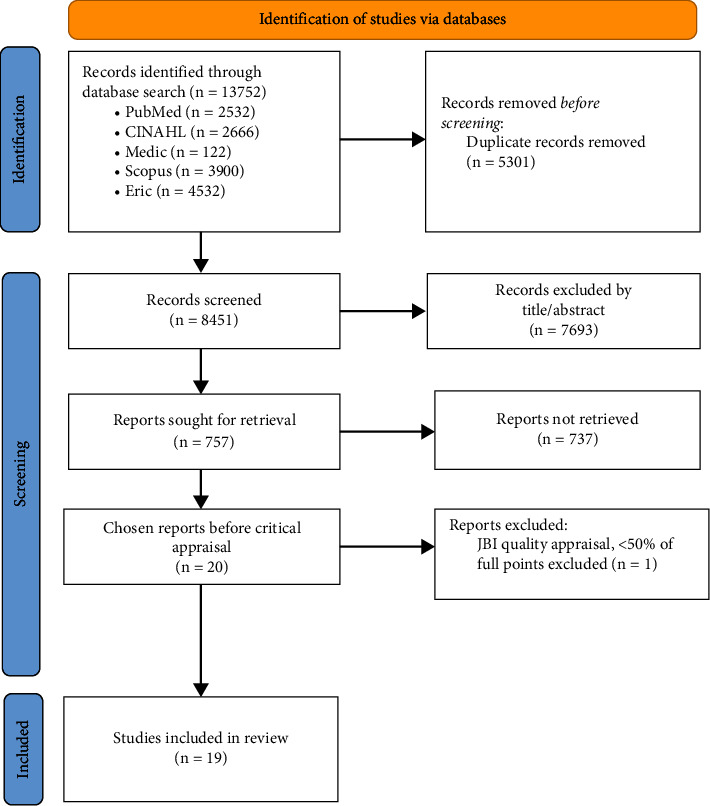
Prisma flow diagram [31].

A total of 13752 publications were retrieved from the database searches (see Figure 1). Six researchers (authors blinded) participated in the screening process. After 5301 duplicate publications were excluded, the total number of studies included was 8451. The next stage involved screening based on titles and abstracts, during which 7694 studies were excluded. Next, full-text screening of *n* = 757 studies was conducted, where 737 papers that did not meet the initial inclusion criteria were eliminated. Each study underwent a double screening, and a third reviewer resolved conflicts. Twenty articles met the inclusion criteria and were subjected to a quality appraisal (see Figure 1).

For quality appraisal assessment, the JBI critical appraisal tool for analytical cross-sectional studies [32] was used to assess the methodological quality of each study (*n* = 20) (see Supplementary File 2). This appraisal tool consists of eight evaluation criteria that examine the methodological quality of an article and determine the extent to which a study has addressed potential biases in its design, conduct, and analysis. Each criterion was evaluated and marked as “yes,” “no,” “unclear,” or “not applicable.” One point was assigned for each criterion rated “yes.” Six researchers (authors blinded) were involved in the quality appraisal assessment to ensure rigorous assessment. Each study underwent a double assessment separately, and disagreements were discussed and resolved through consensus. Inclusion in the review required meeting at least four of the eight requirements, with a total score of at least 50%. Low-quality studies were excluded to maintain the validity of the review's results and recommendations.

Furthermore, 19 studies scored above 50% and were included in the data synthesis. Among these, three studies scored 100%, six scored 75%, seven scored 62.5%, three scored 50%, and one scored 37.5% (excluded from data synthesis). Most of the articles received lower quality scores for various reasons, such as uncertainty regarding the valid and reliable measurement of exposure, lack of clear identification and strategies to address confounding factors, and uncertainty regarding the valid and reliable measurement of outcomes.

### 3.2. Data Extraction and Analysis

The 19 original studies selected were organised by year of publication, country of origin, purpose, methodology (study design, instruments, data collection, and data analysis), participants, and factors associated with the integration and quality assessment score [27] (Table 1). Furthermore, the studies identified and presented the factors most significantly associated with integrating CALD nurses. A narrative synthesis approach was used to synthesise the data [33]. This involved collecting all the narrative results from the selected studies, reducing the data by identifying similarities and dissimilarities, and organising similar findings into meaningful classifications.

## 4. Results

The factors associated with integrating CALD nurses in healthcare organisations were explored in 19 of the original studies. The original studies selected for the systematic review were published between 2008 and 2021 and conducted in the United States (US) (six reviews), United Kingdom (UK) (3), Saudi Arabia (1), Canada (2), Taiwan (4), Korea (1), and Australia (2). The designs of the selected studies were descriptive (*n* = 7), cross-sectional (*n* = 11), and comparative (*n* = 1), and data collection methods that included survey questionnaires and electronic databases were used. The data analysis methods used were statistical parametric analysis and descriptive nonparametric analysis. Participants in the original studies were internationally recruited registered nurses from different countries working as registered nurses in the US, UK, Canada, Australia, Saudi Arabia, and Taiwan. The number of participants varied from 15 to 1951. The systematic review provided an overview of the findings from each study in terms of the factors related to the integration of CALD nurses in healthcare organisations. The data are divided into the following six categories that aimed to answer the research question: (1) sociodemographic characteristics, (2) discrimination, (3) social support, (4) workplace environment, (5) organisational support, and (6) acculturation (see Table 2). The factors influencing integration were classified based on Kamau et al.'s [20] three-dimensional model of integration models and strategies. This model incorporates professional development characteristics, intraorganisational factors, and sociocultural aspects categorised accordingly.

### 4.1. Factors Associated with Professional Development

#### 4.1.1. Career and Competence Development

Statistically significant individual characteristic factors related to the career and competence development of internationally educated nurses (IENs) include their birth year, gender, parenting responsibilities, visible minority status, and level of education. According to Primeau et al. [34]; in a study conducted on the career satisfaction of IENs in Canada, it was found that older and more experienced IENs tended to be more satisfied with their careers compared to their younger or less experienced counterparts (*p* < 0.01). In addition, women expressed higher levels of career satisfaction in the nursing field compared to men (*p* < 0.05). Furthermore, IENs with children under 16 were more satisfied (*p* < 0.05) with their careers than those without parental responsibilities. Men with children reported significantly higher satisfaction levels than women without children (*p* < 0.05). The study also revealed a distinction between visible minority groups (*p* < 0.01), with White and Asian individuals showing significantly higher levels of satisfaction (*p* < 0.05) compared to Black individuals, who tended to be the least satisfied. IENs with nonuniversity degrees before immigrating to Canada exhibited higher career satisfaction (*p* < 0.01) than those with undergraduate degrees, master's degrees, or PhDs. Similarly, the study indicated that higher levels of education attained before immigrating were associated with lower levels of career satisfaction.

Significant differences were observed in education factors in the USA. There was a notable association between IENs and their enrolment in a degree programme after acquiring licensure (*p*=0.01) as well as their pursuit of advanced academic degrees (*p*=0.02) compared to nurses educated in the US (UENs). It was found that twice as many UENs (*n* = 54, 38%) obtained an additional degree following their licensure compared to IENs (*n* = 10, 19%). [35].

Primeau et al. [34] revealed that career characteristics substantially influence career and competence development. It was found that IENs who worked full time showed significantly higher satisfaction levels than those who worked part time or occasionally (*p* < 0.01). In addition, there were notable differences among nursing professions, with registered nurses and registered psychiatric nurses reporting significantly higher satisfaction levels than licensed practical nurses (*p* < 0.01). Furthermore, IENs differed significantly from their host nurses in terms of their practice roles (*p*=0.03), predominantly working as staff nurses (*n* = 52, 98%) with fewer leadership responsibilities (*n* = 1, 2%). Moreover, IENs who believed they had achieved their career goals experienced higher satisfaction levels (*p* < 0.01), while those who faced discrimination expressed lower satisfaction with their careers. The study also highlighted that the first year at the current employer and position had a negative association with career satisfaction. At the same time, there was a moderate and positive association with achieving career goals, all at a significant level of *p* < 0.001. Furthermore, the findings revealed a significant difference in promotions, with UENs reporting a significantly higher frequency of promotions than IENs (*p*=0.04). In comparison to UENs (*n* = 38, 28%), a higher proportion of IENs (*n* = 20, 41%) indicated that they had never received a promotion [35].

Alexis et al.[36] investigated how overseas nurses perceive equal opportunity in the UK. Their study found that African nurses (*p* < 0.001) were more likely to perceive discrimination regarding job refusals based on their ethnic backgrounds. In contrast, Filipino nurses were less likely to have such perceptions. On the other hand, nurses from India and Pakistan had a higher likelihood of being promoted than other groups (*p* < 0.001). In contrast, African nurses were likelier to perceive that they were denied promotions due to ethnicity (*p* < 0.001). Also, geographical location plays a significant role in the perception of ethnic-based denial of promotions (*p* < 0.008).

Primeau et al. [34] found that integration process characteristic factors—a year of immigration (*p* < 0.01), year of the first job (*p* < 0.01), and year of licensure (*p* < 0.01)—were negatively correlated with the job satisfaction of IENs. These results highlight the importance of acculturation and workplace integration with higher levels of job satisfaction among highly qualified immigrants.

When examining organisational characteristics, it becomes evident that IENs working in hospitals experience higher satisfaction levels than those working in long-term care facilities (*p* < 0.05). IENs who perceive themselves as being given fewer opportunities than host nurses or encountering discrimination report significantly lower satisfaction levels (*p* < 0.01). In addition, geographical location plays a role in career satisfaction, with significant variations observed (*p* < 0.01). Furthermore, factors such as mentorship (*p* < 0.01), leadership (*p* < 0.01), promotion (*p* < 0.01), and development (*p* < 0.01) have been identified as significantly correlated with the career satisfaction of IENs [34].

#### 4.1.2. Workplace Mentorship and Precentorship

The study conducted by Adeniran et al. [35] revealed that the level and quality of mentoring received by IENs were deemed insufficient for their advancement to leadership positions compared to that of their counterparts, UENs. Notably, mentors for IENs were found to be more ethnically diverse (*p* < 0.001) and less likely to hold leadership positions within their organisations (*p*=0.01) compared to UEN mentors. In addition, IENs were less inclined to view their mentors as role models (*p*=0.02).

#### 4.1.3. Licensure and Orientation to Work

When examining training factors, Butt et al. [37] investigated the perceived benefits of participation in the Building Bridges Programme among refugee healthcare workers. The Building Bridges Programme is designed to help refugee healthcare workers fill gaps in their cultural, practical, and theoretical knowledge to support them in finding employment. The study findings indicate that among the program participants, 2% could secure registered positions that matched their professional qualifications from their home country, while 41% obtained positions in related healthcare fields. Furthermore, a related study by Covell et al. [38] discovered that specific factors influenced the perceived benefits of participating in a comparable bridging programme. Notably, the classification of the source country as low income (*p* < 0.01) and the IENs having fewer years of professional experience (*p* < 0.01) were associated with a higher perception of benefits from the Bridging Programme. The regression model employed in the study accounted for 11.5% of the variance in the perceived benefits of participating in the Bridging Programme.

The study conducted by Alexis et al. [36] found significant statistical differences in the level of dissatisfaction concerning the number of attended training courses and grades among overseas nurses compared to their white host country counterparts (*p* < 0.001). The research also found disparities in the availability of training course opportunities based on ethnicity and grades (*p* < 0.002).

### 4.2. Factors Associated with Intraorganisational Strategies

#### 4.2.1. Collegial and Peer Support

Alexis's [12] study aimed to investigate the perception of perceived discrimination and ethnicity among international registered nurses (IRNs) in the UK. The findings of the study revealed that IRNs perceived instances of discrimination within the workplace (*p* < 0.001). Specifically, African nurses were more likely to perceive discrimination than nurses from India and Pakistan. In addition, the study highlighted that White British nurses were perceived as exhibiting difficult, aggressive, or hostile behaviour towards IRNs based on their ethnicity (*p* < 0.001).

Furthermore, the study also investigated the perception of social support among IRNs in the UK. It was found that IRNs generally felt supported in their workplace (*p* < 0.001), with both Indian and Pakistani nurses perceiving higher support levels than their international counterparts. On the other hand, African nurses reported receiving the least amount of support in the working environment. Notably, IRNs acknowledged receiving assistance from their White British colleagues, which was statistically significant (*p* < 0.01). Similarly, Alexis et al.'s [36] study supported the findings that experiences of discrimination in the UK varied based on race and ethnicity. Specifically, Black minority IENs were more likely to experience discrimination than Asian-Pacific and Caucasian IENs [12].

#### 4.2.2. Workplace Environment, Diversity, and Employee Treatment

A healthy work environment was found to positively impact the career development of IENs, while poor work environments act as barriers to their career advancement [34, 35]. The research conducted by Goh and Lopez [39] demonstrated that job satisfaction among migrant nurses in Singapore was negatively correlated with the work environment. The study further indicated that international nurses with lower reported acculturation levels also reported lower perceptions of their work environment. Predictors of IENs' intentions to leave their current positions included having supportive nurse managers (*p*=0.03) and a favourable nursing practice environment (*p*=0.01). Also, the study found ethnic differences and Indian nurses reported the highest level of job satisfaction, followed by Malaysian, Filipino, Myanmar, and Chinese nurses. Almansour et al. [40] aimed to investigate the link between nationality and nurse job satisfaction in Saudi Arabia. The study findings indicated that Saudi nurses reported higher levels of satisfaction compared to non-Saudi nurses (IENs) regarding extrinsic rewards (*p* < 0.005) and achieving a work-life balance (*p* < 0.005). Conversely, IENs expressed greater satisfaction than Saudi nurses in areas such as coworker relationships (*p* < 0.005), professional opportunities (*p* < 0.005), and receiving praise and recognition (*p* < 0.005).

The study conducted by Timilsina Bhandari et al. [41] demonstrated that communication in English (*p*=0.001) emerged as the predominant factor associated with job satisfaction among nurses from non-English-speaking backgrounds. In addition, overseas nurses exhibited a negative correlation between the duration of their stay (*p* < 0.05) in Australia and their satisfaction with the work environment. Interestingly, the longer overseas-qualified nurses remained employed in Australia, the less satisfied they became.

Organisational socialisation demonstrated a significant negative correlation with the intention of IRNs to leave within three years (*p* < 0.01). This indicates that IRNs who reported higher levels of organisational socialisation were less likely to leave their current positions within three years. Specifically, two aspects of organisational socialisation—that is, being treated as a colleague by peers (*p* < 0.05) and receiving support from supervisors (*p* < 0.01)—were found to be negatively associated with nurses' intention to leave. Notably, the level of organisational socialisation among IRNs was higher compared to those among other nurse groups, particularly in terms of the item assessing whether the hospital provided adequate orientation (*p* < 0.01) [42].

Geun et al. [43] examined the factors influencing the turnover of Asian foreign-educated nurses in the USA. The findings revealed that perceived quality of orientation (*p* < 0.001) and affective commitment (*p* < 0.001) were significant predictors of turnover at the organisational level. Specifically, the perceived quality of orientation predicted turnover at the organisational level and revealed a trend in predicting turnover at the unit level (*p*=0.01). Additionally, preparatory job search behaviours (*p*=0.04) and active job search behaviours (*p*=0.05) were associated with unit-level turnover.

Alexis [12] study revealed that IENs *perceived discrimination* in the workplace, specifically from patients and their family members (*p* < 0.01). The data further indicated that African nurses were more inclined to perceive such discrimination than the other three groups of IENs. Further, Pittman et al. [44] discovered that IENs experienced discrimination and expressed concerns regarding the disparity in pay (*p* < 0.01) and benefits (*p* < 0.01) compared to their US counterparts. The study revealed that 51% of the IENs reported insufficient orientation, while 40% reported facing at least one discriminatory practice (*p* < 0.01) related to wages, benefits, or shifts/assignments. Compared to other IENs, IENs educated in low-income countries and those recruited through staffing agencies were more likely to report receiving unfair treatment than their US counterparts (*p* < 0.05). In addition, IENs recruited through staffing agencies reported significantly lower wages than self-employed IENs (*p* < 0.05), and the wages were found to be approximately 14% higher for IENs educated in high-income countries compared to those educated in low-income countries.

Liou et al. [45] conducted a study investigating the relationship between acculturation, *collectivist orientation*, and organisational commitment among Asian nurses in hospitals in the US. The findings revealed a significant correlation between collectivism orientation and organisational commitment (*p*=0.001). Participants born in Asian countries other than the Philippines demonstrated lower levels of organisational commitment. In addition, in a study by Cheng and Liou [46], it was discovered that organisational commitment (*p* < 0.001) serves as a significant predictor of the intention of Asian nurses to leave their positions in US hospitals. Moreover, Asian nurses with a stronger collectivist orientation are more willing to embrace the organisation's goals and values, experience higher satisfaction with their current work environment, and display a reduced intention to leave their current job.

The research conducted by Ma et al. [47] demonstrated that Chinese immigrant nurses had a high demand for immigration. It was observed that there was a significant negative relationship (*p*=0.01) between the demands of immigration and the duration of stay in the US. As the duration of stay increased, the demands of immigration decreased, but even among those who had been in the US for over five years, the demands remained relatively high.

#### 4.2.3. Organisational and Management Support and Policies

The study conducted by Alexis et al. [36] found that African IENs, in particular, were unaware of their employers having an equal opportunity policy (*p* < 0.001). In addition, IENs working in London hospitals perceived equal opportunity policies as more effective than nurses in non-London hospitals (*p* < 0.001). Moreover, Filipino nurses expressed a higher likelihood of their skills being utilised than their African counterparts (*p* < 0.002).

### 4.3. Factors Associated with Sociocultural Integration

In a study conducted by Zanjani et al. [48], job satisfaction and integration process characteristic factors were identified as influencing the sociocultural adjustment of IENs to the Australian healthcare system; job satisfaction (*p* <  0.01), current work environment (*p*=0.02), and a sense of feeling at home (*p*=0.01). When IENs achieved a high level of sociocultural adaptation, they reported better overall health and physical well-being. The study also highlights the primary motivations that drove IENs to relocate to Australia. The dominant pull factors were creating a better life for their families (68.5%), improving their financial situation (56.5%), and perceiving political stability (49%) in their new country. Conversely, the main push factors that influenced their decision to leave their home countries were low pay (71.5%) and a lack of opportunities for further nursing education (68%).

According to the findings of Goh and Lopez's [49] study, mainland Chinese IENs working in Singapore exhibited the lowest levels of acculturation. The study also revealed a positive correlation (*p* < 0.01) between acculturation and quality of life, thereby indicating that higher levels of acculturation were associated with a better perception of one's overall well-being. Conversely, a lower perception of the work environment was linked to lower levels of acculturation. In a study by Hayne et al. [50], the researchers examined strategies to help Filipino nurses adapt to cultural aspects after being recruited in the US. The findings indicate that investing in promoting the well-being of recruits in social and work contexts positively impacts job satisfaction and extends to other areas of satisfaction and positive adaptation.

## 5. Discussion

This systematic review identified multiple factors associated with the successful integration of CALD nurses, thereby highlighting their impact on integration strategies and models. These factors were classified into the following six categories: sociodemographic characteristics, discrimination, social support, organisational support, workplace environment, and acculturation.

Numerous factors influence job satisfaction and can vary in cultural contexts and value systems. Low job satisfaction among CALD nurses significantly contributes to high turnover rates, eventually impacting the quality and safety of patient care [51]; [52, 53]. The findings of this review indicate that a range of factors can influence job satisfaction among CALD nurses. The work environment plays a vital role in the job satisfaction and career development of CALD nurses. Factors such as having supportive colleagues, supervisors, and mentors, receiving equal treatment as employees, having access to adequate resources and educational opportunities, and being part of a positive team culture significantly contribute to the overall job satisfaction of CALD nurses. Thus, the findings of this study demonstrate that individual characteristics (age, gender, parenting responsibilities, ethnicity, and education) with factors related to career development, organisational characteristics, and the integration process collectively influence job satisfaction among CALD nurses.

In addition, the findings indicated variations in job satisfaction among different racial and ethnic groups, with Black minority nurses demonstrating lower levels of job satisfaction than their White counterparts. Creating a supportive and inclusive work environment that respects and recognises their characteristics can further enhance the job satisfaction of CALD nurses [51]. When nurses feel valued, respected, and supported in their work environment, they are more likely to experience higher levels of job satisfaction [54, 55].

The findings of this study confirm those of previous research that noted that discrimination against CALD nurses exists in healthcare organisations [11, 56], and minority nurses are at higher risk of discrimination than native or majority nurses [56]. In this study, it was found that CALD nurses may experience differential treatment compared to their colleagues, which includes fewer opportunities for professional development, lower pay and benefits, limited choice in shifts, inadequate access to education, limited chances for promotion and leadership roles, insufficient quality of mentoring, and challenges in maintaining work-life balance. In addition, there was evidence that CALD nurses feel mistreated at work by their fellow nurses, patients, and their families. The experiences of discrimination varied depending on race and ethnicity; those in the Black minority were more likely to experience discrimination and lack of support compared to other CALD nurses and were less unaware of their employers have an equal opportunity policy. Moreover, CALD nurses who were educated in low-income countries or were recruited through staffing agencies were more likely to report experiencing unequal treatment compared to their counterparts—for example, wages were found to be approximately 14% higher for CALD nurses educated in high-income countries compared to those educated in low-income countries.

Furthermore, certain healthcare workplaces and individuals may lack cultural competence, which refers to understanding and effectively working with people from diverse cultural backgrounds [57]. Without this understanding, discrimination and biases can arise, impacting CALD nurses' experiences in the workplace [58–60]. Moreover, inadequate policies, lack of diversity and inclusion initiatives, and biases in recruitment and promotion processes may perpetuate discriminatory practices [60].

A CALD nurse's linguistic competence can challenge their integration into the working environment [61]. This study reveals that communication in English emerged as a predominant factor associated with job satisfaction among nurses from non-English speaking backgrounds. Bridging programmes and language support initiatives are designed to assist CALD nurses in adapting their education and skills to meet the requirements of the new healthcare system. These programs provide language training, cultural orientation, and additional education or training to enhance their competence and enable a smooth transition into the new healthcare environment [62].

Acculturation involves learning and adopting the values, behaviours, and traditions of another group or society, it is the process by which a cultured individual adopts some customs and cultural norms of another culture. This process can happen on a group or individual personal level, for instance, when an individual moves to a new country and adopts the customs of their new cultural context [63]. To enhance the commitment of Asian nurses, it is crucial to understand their cultural values and create a culturally competent and sensitive environment. In the context of Asian cultures, which often have collectivist values, individuals prioritise the needs and goals of the group over individual interests. This orientation can significantly influence the level of organisational commitment among Asian nurses [45, 64, 65]. The findings of this study reveal that Asian nurses with a stronger collectivist orientation demonstrate more significant organisational commitment and job satisfaction. For example, nurses born in China demonstrated a lower level of organisational commitment and acculturation. They expressed a greater desire to immigrate to other countries to practice healthcare. Organisations that employ a significant number of Asian nurses with a collectivist orientation can influence this cultural value by fostering an environment that supports teamwork, collaboration, and a sense of belonging.

Finally, the findings revealed that the factors that influence the sociocultural adjustment of CALD nurses include job satisfaction, the current work environment, and a sense of belonging in the host country. The successful adaptation to the sociocultural aspects of a new country is a crucial component of the migrant experience, thereby impacting mental health and overall psychological well-being in their professional roles [66].

### 5.1. Limitations and Strengths

The PRISMA 2020 checklist was completed and implemented during this systematic review process [31]. One of the limitations of this study concerns publication bias, as it only included published, peer-reviewed articles in English or Finnish, and the search did not include a search for grey literature. In addition, this review was conducted following the JBI guidelines for evidence synthesis, explicitly focusing on systematic reviews to ensure transparency in reporting the review process and findings. In addition, the JBI critical appraisal tool for analytical cross-sectional studies was utilised to assess the methodological quality of the included studies.

The synthesis of statistics in this review posed a significant challenge due to the heterogeneity observed in outcomes across the included studies. Variations in study designs, populations, interventions, and outcomes can impede the pooling of data or the formulation of definitive conclusions.

The adopted methodological choice of a systematic review may have introduced limitations due to the nonconsideration of diverse knowledge, such as policy papers that may have proved valuable to our findings [67]. However, we find that a systematic review was well suited due to the opportunity this research method offers as a systematic, unbiased approach towards providing existing research findings that may inform practice, policy, and future research [68]. Furthermore, systematic reviews are used to build an evidence base that confirms or refutes current practice [28]. In the case of this review, established evidence relating to factors associated with CALD nurse organisational integration may be used to confirm or refute current organisational integration practices.

## 6. Conclusion

The factors associated with integrational strategies and models developed to support the transition and adaptation of CALD nurses to the professional workforce in healthcare include sociodemographic characteristics, discrimination, social support, organisational support, workplace environment, and acculturation. The study highlights the significance of job satisfaction among CALD nurses, emphasising its impact on turnover rates and, consequently, patient care quality and safety. Factors influencing job satisfaction include supportive work environments, equal treatment, access to resources and education, and positive team culture. Furthermore, the review underscores disparities in job satisfaction among different racial and ethnic groups, with Black minority nurses often experiencing lower levels of job satisfaction. It stresses the importance of creating inclusive workplaces to enhance the job satisfaction of CALD nurses. The study also addresses discrimination against CALD nurses within healthcare organisations, noting challenges such as limited professional development opportunities, unequal pay, and mistreatment by colleagues, patients, and families. In addition, it discusses the role of cultural competence in CALD nurse integration, highlighting the importance of communication skills, language support initiatives, and understanding cultural values, particularly among Asian nurses with collectivist orientations. Finally, the review emphasises the impact of sociocultural adjustment on CALD nurses' professional roles and overall psychological well-being, stressing the importance of support mechanisms for successful adaptation.

Overall, this systematic review provides comprehensive insights into the challenges and facilitators of integrating CALD nurses into healthcare systems, offering valuable implications for policy and practice in fostering inclusive and supportive work environments. This research is valuable for identifying specific integration needs and adapting support strategies accordingly. Based on the study's outcomes, we recommend policymakers, nurse employers, and nurse leaders implement targeted interventions, engage CALD nurses in ongoing professional development, and provide language support services to improve the supportive environment. Comprehensive cultural competency training for all healthcare staff, including managers, enhances cultural competence in healthcare work environments, improving the ability to work with nurses from diverse cultural backgrounds effectively. Establishing clear guidelines to address discrimination and bias creates a supportive environment where CALD nurses feel valued and respected, facilitating their adaptation and integration into the healthcare organisation. In future research, there is a need to address the worsening global nursing shortage, which is driving a rise in international nurse migration to developed countries. Ensuring fair treatment and ethical integration is crucial for CALD nurse work satisfaction and organisational success. There is a need to involve patients through research and understand their experiences with CALD nurses, which may help better patient-CALD nurse relations and may result in positive care outcomes. With emerging technology-enhanced healthcare as a solution for nurse human resource shortages, patients uncomfortable with international nurses may choose technology as a substitute. This raises the question of whether developed countries' healthcare institutions will invest in international nurse integration due to cost concerns. We, however, note that nurse migration is on the rise due to current and future nursing workforce shortages, and research on organisational integration of CALD nurses shows that better outcomes could be achieved if organisations were to invest and structure integration strategies within the formal structure of a healthcare organisation.

## 7. Relevance to Clinical Practice

Our results point out factors that associate integrational strategies and models to support the transition and adaptation of CALD nurses to the professional workforce in healthcare settings. Ensuring the integration of CALD nurses into clinical practice benefits encompass enhancing of diversity and cultural competence of the healthcare team and enabling knowledge and skills exchange with nurses who have a global health perspective prevalent in their home countries. Cultural diversity enhances patient-centered care by making patients feel more comfortable and respected when coming from diverse backgrounds themselves. Eventually, CALD nurses' integration can address workforce shortage and competence exchange among the countries. This study has a significant implication on nursing management since previous research on CALD nurse organisational integration has established that nurse leaders and managers are integral in supporting the entire workforce through equity and equality towards bettering CALD nurse integration. The findings have established factors associated with CALD nurse organisational integration; these findings impact nursing workforce practices and how a healthcare organisation may invest in developing structural strategies and models that support CALD nurse integration the best.

## Figures and Tables

**Table 1:** Data extraction.

Author(s), year of publication, country	Purpose	Methodology: study design, instruments, data collection, data analysis	Participants	Factors associated with integration	Quality assessment
Adeniran et al., 2013, USA	To determine differences between internationally educated nurses and nurses educated in the United States in their levels of mentoring functions, self-efficacy, and participation in professional development and career advancement	A descriptive design, cross-sectional (1) Mentorship measure. (2) New General Self-Efficacy Scale. (3) Demographic questionnaire A web-based survey A power analysis, descriptive statistics, frequency and percentage estimates for categorical variables, mean, standard deviations, *t*-tests, Chi-square analyses	*n* = 200 registered nurses (educated in the United States *n* = 145, internationally educated nurses *n* = 55), age 22–65 years, currently working in hospital settings for a minimum of 3 years within Philadelphia County	The level and quality of mentorship functions received by internationally educated nurses (IENs) were insufficient for them to advance to leadership positions as their counterparts' nurses educated in the United States (UEN). Significant disparities were noted in the role model function of mentoring (*p*=0.02). Mentors for IENs were more ethnically diverse and less likely to hold leadership positions in their organisations. IENs (*n* = 10.18%) were half as likely to pursue another degree compared with UENs (*n* = 51.36%). UENs were significantly different from IENs in their practice role (*p*=0.03). They reported receiving promotions significantly more frequently than IENs (*p*=0.04). IENs worked predominantly as staff nurses (*n* = 52.98%), with a mere one IEN reporting working in the area of leadership (*n* = 1.2%). Practice roles among UENs were more diverse (*n* = 28.21%)	8
Alexis, 2014, UK	To determine internationally registered nurses' perception of discrimination, support, and their adjustment to a new environment in the National Health Service in England	A descriptive design. (1) Discrimination. (2) Support. (3) Adjustment to a new environment A paper survey was constructed following the emergent themes from the qualitative data analysis Chi-square, Fisher exact, Kruskal–Wallis test	*n* = 188 internationally recruited registered nurses from 15 National Health Service Hospitals in England who be working for a minimum of 1 year, Black or of minority ethnic origin and had to be qualified as an international nurse	International registered nurses (IRNs) perceived that they were discriminated against in the workplace (*p* < 0.00) and patients and family members behaved difficultly and aggressively toward them (*p* < 0.00). IRNs perceived that White British nurses were aggressive towards them for a reason based on their racial features (*p* < 0.01). IRNs felt supported in their workplaces (*p* < 0.01). IRNs from Africa perceived discrimination as evident in the workplace; the support they received was limited, and their adjustment to a new environment was the weakest compared to the other IRNs	4

Alexis and Vydelingum, 2009, UK	To determine how overseas nurses perceive equal opportunity as well as the opportunities for skill development and training to be in the National Health Service in the United Kingdom	A descriptive design, a questionnaire approach. (1) Biographical information for example grade. (2) Years of experience in their country of origin and the national health service. (3) Equal opportunity. (4) Discrimination. (5) Support mechanisms. (6) Adjustment to a new environment. (7) Skill development and training A survey approach was adopted to investigate the aims of the study A simple descriptive statistics, Chi-squared tests, Fisher's exact tests, Kruskal–Wallis tests, Mann–Whitney *U* tests, Spearman's tests	*n* = 188 registered nurses and qualified as overseas nurses, black and minority ethnic origin, and be working in the National Health Service in the United Kingdom for a minimum of one year	Overseas nurses from African nurses perceived that were refused jobs based on their ethnic backgrounds whereas Filipino nurses were less likely to perceive this. Nurses from India and Pakistan were more likely to be promoted than any other group of international nurses. African nurses were more likely to perceive that they had been refused promotion based on their ethnicity. Filipino nurses indicated that their skills were more likely to be used than those of their African counterparts. In addition, the survey revealed that overseas nurses employed in NHS hospitals in London were more likely to be promoted and supported and less likely to have aggressive behaviour directed at them compared to those in NHS hospitals in non-London regions	5
Almansour et al., 2020, Saudi Arabia	To investigate whether there is an association between nationality and nurse job satisfaction	A cross-sectional design (1) McCloskey/Mueller Satisfaction Scale An online survey and a paper survey Preliminary analysis, a multiple linear regression analysis, a descriptive analysis	*n* = 743 nurses from three major Government Hospitals in Saudi Arabia	Non-Saudi nurses had lower satisfaction rewards such as pay, holiday entitlement, and work/life balance. Compared with Saudi nurses, expatriate nurses had overall lower job satisfaction after controlling for other predictors. Expatriates were less satisfied than Saudi nurses with extrinsic rewards and family-work balance. However, Saudi nurses were less satisfied with their professional opportunities, praise and recognition, and coworker relationships	8
Bae, 2011, USA	To examine international nurses' perceptions of their organizational socialization and its association with intent to leave in both international and American nurses	A descriptive design, secondary analysis of data from a hospital registered nurses survey (1) Organizational socialization (the quality of the orientation programme and support from supervisors and peers). (2) Nurses' intent to leave (within three years). (3) Nurses' country of origin A paper survey, an online survey Analysis of variance and Chi-square tests, a logistic regression model	*n* = 752 registered nurses (*n* = 245 international registered nurses, *n* = 507 American registered nurses) in the greater New York metropolitan area with less than five years of registered nurse experience in the USA	The orientation programme and support from peers and supervisors played an important role in the international nurse's organizational socialization process. Good supervisor and peer support were negatively associated with nurses' intent to leave (i.e., these nurses were less likely to leave within three years). The level of organizational socialization of foreign-educated RNs was higher than that of any other nurse groups, especially when looking at the item entitled “hospital provided adequate orientation” (*p* < 0.01). Lower proportions of the foreign-educated RNs (26%) and adult immigrant RNs (29.1%) reported that they had plans to leave within three years compared to American RNs (45.2%) and child immigrant RNs (39.3%)	6
Butt et al., 2019, UK	To describe the employment outcomes of a refugee healthcare professional who participates in the Building Bridges Programme in the United Kingdom National Health Service	A comparative design, statistical and contractual reporting (1) Employment outcomes. (2) (%) proportion of refugee healthcare professionals joining the Building Bridges Programme who settle in an associated healthcare profession position An electronic database Statistical and contractual reporting	*n* = 83 refugee nurses who participated in the Building Bridges Programme from October 2009 to March 2018 and sought employment in the UK National Health Service	The Building Bridges Programme provides 2/83 (2%) nurses settled into a registered National Health Service position appropriate to their (home country) professional qualifications. 34/83 (41%) nurses settled in associated healthcare profession positions	4
Cheng and Liou, 2010, Taiwan	To measure the predictability of cultural orientation on organisational commitment, perception of practice environment, and intention to leave amongst Asian nurses working in US hospitals	A cross-sectional design, postsurvey. (1) The Organisational Commitment Questionnaire. (2) Practice Environment Scale of the Nursing Work Index. (3) Anticipated Turnover Scale. (4) Collectivist Orientation Scale with satisfactory reliability A postal survey descriptive statistics, hierarchical regression, Pearson correlation, Mann–Whitney *U*-test, Sobel-test	*n* = 195 Asian nurses (44.1% Filipinos, 32.8% Chines) working at least six months in US hospitals	Organisational commitment is a key predictor of Asian nurses' intention to leave. Asian nurses who are more collectivist-oriented are more willing to accept the goals and values of the organisation, exert effort on behalf of the organisation, are more satisfied with their current practice environment and have less intention to leave their current job	5
Covell et al., 2018, Canada	To examine internationally educated nurses' perceptions of the extent to which participating in bridging programmes is beneficial for preparing to practise nursing in Canada	A cross-sectional design (1) Demographics. (2) Perceived benefits of bridging program participation (B^2^P^2^)-scale descriptive statistics, linear multiple regression analysis	*n* = 360 internationally educated nurses who participated in bridging programmes, and live and work permanently as a nurse in Canada	Bridging programmes help internationally educated nurses address gaps in their cultural, practical, and theoretical knowledge. Source country and amount of professional experience influence the extent to which internationally educated nurses benefit from participating in bridging programmes in Canada. The regression model explained 11.5% of the variance in perceived benefits of bridging programme participation. Two predictors were statistically significant: source country and professional experience	8
Geun et al., 2018, Korea	To investigate factors affecting the turnover of Asian Foreign-educated nurses	A cross-sectional design (1) Supplement digital content 1 and 2. (2) Organizational Commitment Questionnaire. (3) McCain and Marklin Social Integration Scale. (4) Confidence and communication. (5) Job search behaviours instrument. (6) General self-rated health instrument: an online survey backward multivariable logistic regression	*n* = 201 Asian foreign-educated nurses in their 1^st^ year of employment in the United States	Perceived quality of orientation and affective commitment were the only significant predictors of turnover at the organizational level of Asian foreign-educated nurses. Perceived quality of orientation predicted organizational-level turnover and trended toward predicting unit-level turnover	6
Goh and Lopez, 2015, Singapore	To examine the acculturation level of international nurses working in a multicultural society. The relationship between acculturation, working environment, and quality of life of international nurses was also explored	A cross-sectional, correlational study (1) World Health Organisation Quality of Life-BREF. (2) Practice Environment Scale of the Nursing Work Index-Revised A paper self-report questionnaire Descriptive statistic, histogram and QQ plot, mean score and deviation, a Pearson product-moment correlation coefficient	*n* = 814 international nurses working in Singapore	There were variations in the acculturation level among different nationality groups of international nurses. Acculturation levels were the lowest among mainland Chinese international nurses (*M* = 27.47, SD 5.23). A positive correlation was found between acculturation and quality of life whereas a lower perception of the work environment was associated with a lower acculturation level	6
Goh and Lopez, 2016, Singapore	To explore the job satisfaction level of migrant nurses working in a multicultural society and the relationship between their job satisfaction levels, work environment, their intentions to leave, and the predictors of their intentions to leave	A cross-sectional, correlation design using a stratified random sample (1) A demographic sheer. (2) The job satisfaction questionnaire (JSQ). (3) The practice environment scale-nursing work index-revised A survey A histogram prior, descriptive statistic, mean and standard deviations, a Pearson correlation coefficient analysis	*n* = 495 migrant nurses working in a tertiary public-funded hospital in Singapore for at least one year	The presence of a supportive work environment is essential to retain migrant nurses. The results showed that migrant nurses were satisfied with their jobs, with job satisfaction negatively correlated with the work environment. Pre-existing groups of Chinese migrant nurses did not help newly arrived Chinese migrant nurses assimilate better. Predictors of migrant nurses' intentions to leave included having supportive nurse managers and a nursing practice environment	5
Hayne et al., 2009, USA	To examine strategies to facilitate the cultural adaptation, job satisfaction, and perception of role and social support of a group of recruited Filipino nurses	A descriptive design (1) The nursing work index-revised edition. (2) Occupation stress inventory-revised edition A survey Normative statistic	*n* = 15 Philippine nurses who were recruited to the USA in 2003 and 2004	Investment in promoting the well-being of recruited nurses, as illustrated by the significant planning effort and strategies employed by this organization, pays off in job satisfaction, and spills over into other areas of satisfaction and positive adaptation. Investing in promoting the well-being of recruits in both social and work contexts positively benefits job satisfaction and spills over into related areas of satisfaction and positive adaption	6
Liou et al., 2013, Taiwan	To examine the relationship between acculturation, collectivist orientation, and organisational commitment among Asian nurses in US hospitals	A cross-sectional design using snowball sampling (1) The collectivist orientation scale. (2) Organisational commitment questionnaire. (3) Acculturation factors A paper survey A power analysis Pearson correlation, ANOVA, and regression	*n* = 195 east Asian nurses working in hospitals across the United States at least six months and performing direct patient care	To increase Asian nurses' commitment, administrators must understand their cultural values and provide them with a culturally competent and sensitive environment. Participants scored high on collectivism and commitment. Collectivism was significantly correlated with commitment but did not mediate acculturation factors and commitment	6
Liou and Grobe, 2008, Taiwan, Texas	To examine the relationship among collectivist orientation, perception of practice environment, organizational commitment, and intention to leave	A cross-sectional, correlational design snowball sampling (1) Collectivist Orientation Scale. (2) The Practice Environment Scale of the Nursing Work Index. (3) The Organizational Commitment Questionnaire. (4) Anticipated Turnover Scale Questionnaire Descriptive statistics, Pearson correlation and regression	*n* = 35 Asian nurses work in U.S. Hospitals	To prevent Asian nurses from leaving employment settings, increasing their organizational commitment appears to be indicated. Because perception of the practice environment is an antecedent of organizational commitment, providing a practice environment where nurses are satisfied is an alternative strategy to retain nurses. Organizational commitment mediates the perception of the practice environment and the intention to leave	5
Ma et al., 2010, USA	To identify the demands of immigration among Chinese nurses who have immigrated to the USA. The relationship between the demands of immigration and length of stay in the USA was also investigated	A descriptive correlational study design (1) The demands of immigration scale. (2) Demographic questionnaire A self-administered survey frequency distributions, range, descriptive statistics	*n* = 128 Chinese nurses and immigrated to the USA	The immigration demands decreased as the length of stay in the USA increased. Still, the demands of immigration levels remained high for Chinese immigrant nurses compared to the Indian and Filipino nurses. Chinese immigrant nurses have high demands for immigration. There was a significant negative relationship between the demands of immigration and the length of stay in the USA. Immigration demands decreased as the length of stay increased but remained high even for those who had been in the USA for >5 years	5
Pittman et al., 2014, USA	To determine whether foreign educated nurses perceived they were treated equitably in the U.S. workplace during the last period of high international recruitment from 2003 to 2007	A descriptive design Four outcomes of interest. (1) Hourly wages. (2) Job satisfaction. (3) Adequacy of orientation. (4) Perceived discrimination An online survey Descriptive and regression analysis	*n* = 629 foreign-educated nurses in the USA	Foreign-educated nurses educated in low-income countries and those recruited by staffing agencies were significantly more likely than other foreign-educated nurses to report that they receive inequitable treatment compared with their U.S. counterparts. 40% of the foreign-educated nurses in this study perceived their wages, benefits, or shift or unit assignments to be lower than those of their American colleagues. Respondents from high-income countries were significantly less likely to perceive discrimination than respondents from low-income countries. 51% of respondents reported receiving insufficient orientation and 40% reported at least one discriminatory practice regarding wages, benefits, or shift or unit assignments	4
Primeau et al., 2021, Canada	To identify the main correlates of internationally educated nurses' career satisfaction	A cross-sectional analysis The instrument developed for the study includes four sections. (1) Eligibility (2) Integration. (3) Career advancement. (4) Demographics A survey electronically or on paper Kruskal–Wallis test, Spearman rank correlation test, and Mann–Whitney *U* test	*n* = 1951 internationally educated nurses in Canada	Older and more experienced internationally educated nurses tended to be more satisfied with their careers than their younger or less experienced colleagues were. Males were inclined to be less satisfied than their female counterparts, and having children tended to make all three groups more satisfied. The higher level of education before immigrating the lower the career satisfaction. As for organizational characteristics, full-time nurses were more satisfied than those working part time or with occasional employment. Career satisfaction varied greatly depending on sociodemographic characteristics, organizational settings, and geographic location. Internationally educated nurses who identified as White or Asian had the highest level of career satisfaction, whereas those who identified as Black tended to be the least satisfied. Internationally educated nurses who thought they had achieved their career goals were more satisfied, while those who experienced discrimination were less satisfied with their careers	5

Timilsina Bhandari and Xiao, 2014, Australia	To explore factors associated with the job satisfaction of overseas qualified nurses working in public hospitals in South Australia and to compare whether factors associated with job satisfaction of overseas nurses from English-speaking backgrounds differed from those from non-English-speaking backgrounds	A cross-sectional survey design (1) Job satisfaction of overseas-qualified nurses: index of work satisfaction, nursing work index-R, Mueller, and McCloskey satisfaction scale A survey Kolmogorov–Smirnov test, A Mann–Whitney *U*-test, Chi-square test, Spearman's correlation, content analysis	*n* = 151 overseas qualified nurses who work in five major public hospitals in South Australia	Four factors were found to influence job satisfaction: a supportive work environment, interpersonal relationships, communication in English, and salary and salary-related benefits. Communication in English was the predominant factor that was associated with job satisfaction in nurses from non-English-speaking backgrounds. This group of nurses also showed a negative correlation between length of stay in Australia and satisfaction with their work environment. Participants' responses to open-ended questions revealed issues relating to discrimination and racism	5
Zanjani et al., 2020, Australia	The primary aim was to examine factors associated with overseas qualified nurses' sociocultural adjustment to the Australian healthcare system. A secondary aim was to determine whether there was a correlation between overseas qualified nurses' sociocultural adjustment and their mental and physical health	A cross-sectional study (1) Sociocultural adaptation scale-revised. (2) The nurse international and transition questionnaire-2. (3) The perceived stress scale and general health questionnaire-12 A survey electronically Linear regression analysis, the Pearson correlation	*n* = 200 overseas qualified nurses working as registered nurses in the Australian healthcare system. Participants' English was not their first language and had completed the bridging courses offered in Australia before being granted nursing registration	Job satisfaction and feeling supported in the workplace are the most important factors influencing OQNs' successful adjustment into the Australian healthcare system. Three factors (job satisfaction, current work environment, and feeling at home in Australia) were found to be significant in measuring OQN's level of sociocultural adaptation. When the level of sociocultural adaptation was high, OQNs reported better general and psychological health	6

**Table 2:** Factors associated with integrational strategies and models to support CALD nursing staff transition and adaptation to the professional workforce in healthcare settings.

Factors	Outcomes						
	Professional development			Intra-organizational			Sociocultural
	Career and competence development	Workplace mentorship and precentorship	Licensure and orientation to work	Collegial and peer support	Workplace environment, diversity, and employee treatment	Organisation and management support and policies	Cultural training, learning and support
Participants (*n*)	*n* = 2339	*n* = 200	*n* = 393	*n* = 388	*n* = 2018	*n* = 1336	*n* = 200
Sociodemographic characteristic							
Individual characteristic							
Year born	**p** < 0.01						
Gender	**p** < 0.05				**p** < 0.05		
Parenting	**p** < 0.05						
Visible minority	**p** < 0.01						
Education	**p** < 0.01						
Education							
Continued education credits/year	*p*=0.08						
Received formal degree since last education	**p**=0.01						
Currently pursuing an academic degree	**p**=0.02						
Professional certification completed	*p*=1.00						
Career characteristic							
Work status	**p** < 0.01						
Work hours/week	*p* < 0.50						
Hourly income	*p* < 0.30						
Annual income	*p* < 0.06						
Practice area	*p* < 0.60						
Practice role	**p** < 0.03						
Pay type	*p* < 0.28						
Nursing profession	**p** < 0.01						
First-year current employer	**p** < 0.01						
First-year current position	**p** < 0.01						
Achievement career goals	**p** < 0.01						
Integration process characteristic							
Year of migration	**p** < 0.01						
Year of the first job	**p** < 0.01						
Year of licence	**p** < 0.01						
Feeling at home							**p**=0.01
Organisational characteristic							
Work setting	**p** < 0.05						
Region	**p** < 0.01						
Mentorship	**p** < 0.01						
Leadership	**p** < 0.01						
Promotion	**p** < 0.01						
Development	**p** < 0.01						
Collectivist orientation					**p**=0.01		
Organisational commitment					**p** < 0.001		
Discrimination							
Experienced discrimination in the workplace	**p** < 0.01			**p**=0.001			
Aggressive patients and their relatives					**p**=0.001		
Aggressive White British nurses				**p** < 0.001			
Believe that refused jobs based on their ethnicity	**p** < 0.001						
Average hourly wage					**p** < 0.05		
Average job satisfaction score					NS		
Adequacy of orientation					NS		
Average count of perceived discriminatory practices						**p** < 0.05	
Perceived at least on discriminatory practice						**p** < 0.01	
Believed they did not receive pay comparable to U.S. peers					**p** < 0.01		
Believed they did not receive the same benefits as U.S. peers					**p** < 0.01		
Believed they received fewer desirable shifts or units than U.S. peers					NS		
Believed that refused jobs based on their ethnicity						**p** < 0.001	
The relationship between equal opportunity policies and ethnicity						**p** < 0.001	
The relationship between equal opportunity and organisations						**p** < 0.001	
The relationship between skills acquired from overseas and ethnicity						**p** < 0.001	
Social support							
Social support		*p*=0.90					
Feeling supported and ethnicity				**p**=0.001			
Adjusting to a new environment with assistance from White British nurses and ethnicity				**p** < 0.01			
Treated as a colleague by peers					**p** < 0.05		
Supported at work by supervisors					**p** < 0.01		
Organisational support							
Promotion							
Last promotion through a career ladder	**p**=0.04						
Applied for promotion	**p** < 0.001						
Success in getting the promotion after applying	**p** < 0.001						
The relationship between refused promotion and organisations	**p** < 0.001						
Bypassed for promotion and racial features	**p** < 0.001						
Bypassed for promotion and organisations	**p** < 0.001						
Mentoring							
Mentoring functions				*p*=0.90			
Role model		**p**=0.02					
Self-efficacy		*p*=0.90					
Mentor's profile							
Mentor's sex		*p*=0.30					
Mentor's position		**p**=0.01					
Mentor's race	*p*=0.80	**p**=0.01					
Training							
Dissatisfied with the number of training courses						**p** < 0.001	
Lack of opportunity to go on training courses based on colour or race						**p** < 0.001	
Encouragement by managers to attend training courses						**p** < 0.001	
Nursing training in a non-English language							
Years completed nursing training							*p*=0.76
Given sufficient time to become acquainted with the methods and procedures of their working environments						**p** < 0.001	*p*=0.12
Workplace environment							
Job satisfaction							
Current work environment					*p*=0.46		**p** < 0.01
Supportive work environment					*p*=0.35		**p**=0.01
Interpersonal relationship					*p*=0.39		
Communication in English					**p**=0.001		
Salary and salary-related benefits					*p*=0.54		
Extrinsic rewards					**p** < 0.001		
Scheduling					*p*=0.06		
Family/work balance					**p** < 0.001		
Coworkers					**p** < 0.001		
Interaction opportunity					*p*=0.62		
Professional opportunity					**p** < 0.001		
Praise/recognition					**p** < 0.001		
Control/responsibility					*p*=0.95		
Years of experience					**p**=0.02		
Length of stay					**p** < 0.05		
Salary					*p*=0.26		
Education					*p*=0.59		
Nationality							
Filipino	*p*=0.22						
Indian	*p*=0.39						
Jordanian	*p*=0.12						
South African	**p** < 0.05						
Malaysian	*p*=0.81						
British	**p**=0.05						
Other	**p** < 0.05						
Acculturation							
Perceived benefits of bridging program participation							
Source country: low income			**p** < 0.01				
Language proficiency			*p*=0.45				
Professional experience: years			**p** < 0.01				
Academic preparation: baccalaureate			*p*=0.27				
Academic preparation: graduate			*p*=0.52				
Settled into a registered NHS position appropriate to their home country			2%				
Settled in associated healthcare profession positions			41%				
Intention to leave							
Nurse's intent to leave within 3 years;							
Age					**p** < 0.01		
Gender					**p** < 0.01		
Citizenship					*p*=0.23		
Marital status					*p*=0.10		
Religion					*p*=0.14		
Nursing qualifications					*p*=0.81		
Work experience out of Singapore					*p*=0.85		
Years of experience					*p*=0.13		
Nursing practice environment					*p*=0.40		
Quality care					*p*=0.49		
Nurse manager ability					**p**=0.01		
Autonomy/professionalism					*p*=0.12		
Employment characteristics and demographics					**p**=0.03		
Treated as a colleague by peers					**p**=0.06		
Supported at work by supervisors					**p** < 0.01		
The hospital provided adequate orientation					**p** < 0.01		
Turn over organizational level							
Orientation evaluation						**p**=0.001	
Language fluency						*p*=0.09	
Affective organisational commitment						**p**=0.001	
Continuance organisational commitment						*p*=0.64	
Normative organisational commitment						*p*=0.34	
Social support						*p*=0.72	
Preparatory job search behaviour						*p*=0.15	
Active job search behaviour						*p*=0.127	
Self-rated health-poor						*p*=0.13	
Turn over unit level							
Orientation evaluation						**p**=0.04	
Language fluency						*p*=0.19	
Affective organisational commitment						**p**=0.01	
Continuance organisational commitment						*p*=0.82	
Normative organisational commitment						*p*=0.82	
Social support						*p*=0.11	
Preparatory job search behaviour						**p**=0.04	
Active job search behaviour						**p**=0.05	
Self-rated health: poor						*p*=0.23	

Statistical significance has been marked in bold, *p* values <0.05.

## Data Availability

All data generated during this study are included within the article.

## Additional Points

*What Does This Paper Contribute to the Wider global Community?* (i) Effective integration allows culturally and linguistically diverse nurses to maximise their career potential as professional nurses in their host countries. Therefore, any strategies and models developed and implemented must adopt a multidimensional approach, considering factors associated with integrating culturally and linguistically diverse nurses. (ii) A safe and supportive work environment plays a vital role in the job satisfaction and career development of culturally and linguistically diverse nurses. (iii) Black minority nurses demonstrate lower levels of job satisfaction than their White counterparts, emphasising the need to build an inclusive work environment with zero avoidance to discrimination.

## Supplementary Materials

Supplementary Materials Supplementary File 1: search strategy used in the electronic databases. Supplementary File 2: assessment of methodological quality of the included studies using JBI critical appraisals.
