# Supplementary material for: Factors Associated with the Integration of Culturally and Linguistically Diverse Nurses into Healthcare Organisations: A Systematic Review of Quantitative Studies
Source: J Nurs Manag. 2024 Jun 24;2024:5887450. doi: 10.1155/2024/5887450 (PMC11918648; doi:10.1155/2024/5887450)
Supplement: Supplementary Materials — Supplementary File 1: search strategy used in the electronic databases. Supplementary File 2: assessment of methodological quality of the included studies using JBI critical appraisals. [file 5887450.f1.docx]

Supplementary file 1. Search strategy used in the electronic databases

| *PubMed* | ((((((("Nurses"[Mesh]) OR Practical Nurses"[Mesh]) OR "Nursing,  Supervisory"[Mesh]) OR "Faculty, Nursing"[Mesh]) OR (((nurs*[Text Word]) AND  (lead*[Text Word] OR director*[Text Word] OR executive*[Text Word] OR manager*[Text Word] OR supervis*[Text Word] OR administrator*[Text Word] OR specialist*[Text Word]  OR midwi*[Text Word] OR teacher*[Text Word] OR educator*[Text Word] OR facult*[Text  Word])) OR (Nurse*[Text Word] OR midwi*[Text Word]))) NOT (student*[Text Word])) AND (((((("Social Integration"[Mesh]) OR "Personnel Selection"[Mesh]) OR "Inservice Training"[Mesh]) OR "Social Inclusion"[Mesh]) OR "Acculturation"[Mesh]) OR (integration*[Text Word] OR recruitment[Text Word] OR "Personnel selection"[Text Word] OR orientation[Text Word] OR "Inservice training"[Text Word] OR inclusion[Text Word] OR acculturation[Text Word] OR adaptation[Text Word] OR transition[Text Word] OR retention[Text Word] OR adjustment[Text Word] OR "organizational socialization"[Text Word] OR onboarding[Text Word] OR newcomer*[Text Word] OR "organizational entry"[Text Word]))) AND ((("Cultural Diversity"[Mesh]) OR "Emigration and Immigration"[Mesh]) OR ("cultural divers*"[Text Word] OR "linguistically divers*"[Text Word] OR "culturally divers*"[Text Word] OR "linguistic divers*"[Text Word] OR "culture divers*"[Text Word] OR cald[Text Word] OR immigra*[Text Word] OR foreign*[Text Word] OR international*[Text Word] OR oversea*[Text Word])) |
| --- | --- |
| *CINAHL* | ( ( (MH "Nurses+") OR (MH "Nursing Management+") OR (MH "Midwives+") ) OR ( ( nurs* AND ( lead* OR director* OR executive* OR manager* OR supervis* OR administrator* OR specialist* OR midwi* OR teacher* OR educator* OR facult* ) ) OR ( nurse* OR midwi* ) NOT student* )  AND  ( ( (MH "Social Integration") OR (MH "Personnel Selection+") OR (MH "Social Inclusion") OR (MH "Acculturation") ) OR ( integration* OR recruitment OR "Personnel selection" OR orientation OR "Inservice training" OR inclusion OR acculturation OR adaptation OR transition OR retention OR adjustment OR "organizational socialization" OR onboarding OR newcomer*  OR "organizational entry" ) )  AND  ( ( (MH "Cultural Diversity") OR (MH "Emigration and Immigration") ) OR ( "linguistic* divers*" OR "cultur* divers*" OR international* OR oversea* OR cald OR immigra* OR foreign* OR international* OR oversea* ) ) |
| *Medic* | nurs* midwi* hoitaj* sairaanhoitaj* hoitohenkilö* kätilö* osastonhoitaj*  AND  "Social Integration" "Personnel Selection" "Inservice Training" Acculturation "Social  Inclusion" rekrytoi* inkluusi* valin* sopeutu* orientoitu* oppimi* koulut*  AND  "Cultural Diversity" "Emigration and Immigration" kansainväli* ulkoma* monimuotoi* maahanmuutt* etni* kulttuur* |
| *Scopus* | ( TITLE-ABS-KEY ( ( nurs* AND ( lead* OR director* OR executive* OR manager*  OR supervis* OR administrator* OR specialist* OR midwi* OR teacher* OR educator* OR facult* ) ) OR nurse* OR midwi* ) AND TITLE-ABS-KEY ( "linguistic* divers*" OR  "cultur* divers*" OR international* OR oversea* OR cald OR immigra* OR foreign*  OR international* OR oversea* ) AND TITLE-ABS-KEY ( integration* OR recruitment OR "Personnel selection" OR orientation OR "Inservice training" OR inclusion OR acculturation OR adaptation OR transition OR retention OR adjustment OR "organizational socialization" OR onboarding OR newcomer* OR "organizational entry" ) ) AND ( LIMITTO ( LANGUAGE , "English" ) OR LIMIT-TO ( LANGUAGE , "Finnish" ) ) |

Search strategy

"Licensed

*ProQuest* ((nurs* AND (lead* OR director* OR executive* OR manager* OR supervis* OR administrator* OR specialist* OR midwi* OR teacher* OR educator* OR facult*)) OR nurse* OR midwi*) AND ("linguistic* divers*" OR "cultur* divers*" OR international* OR oversea* OR cald OR immigra* OR foreign* OR international* OR oversea*) AND (integration* OR recruitment OR "Personnel selection" OR orientation OR "Inservice training" OR inclusion OR acculturation OR adaptation OR transition OR retention OR adjustment OR "organizational socialization" OR onboarding OR newcomer* OR "organizational entry") + kielirajaukset + peer reviewed + Show results outside my library's subscription

**Supplementary File 2.** Assessment of methodological quality of the included studies

| JBI Critical  Appraisal Checklist for Analytical Cross-Sectional  Studies | Adeni-  ran et al.  2013 | Alexis  2014 | Alexis  & Vydenigum 2009 | Alma  n-sour et al.  2020 | Bae  2012 | Butt et al.2019 | Chen g & Liou  2011 | Covell et al. 2018 | Geun et al.  2018 | Goh &  Lopez  2016a | Gog &  Lopez 2016b | Hayn  e at al.  2009 | Liou  &  Grobe  2008 | Liou et al.  2013 | Ma et al.  2010 | O’Brie n-  Pallas  &  Wang  2006 | Pittman et al.  2014 | Primeau  et al. 2021 | Timilsin a et al.  2014 | Zanjani et al. 2021 |
| --- | --- | --- | --- | --- | --- | --- | --- | --- | --- | --- | --- | --- | --- | --- | --- | --- | --- | --- | --- | --- |
| Were the criteria for inclusion in the sample clearly defined? | Yes | Yes | Yes | Yes | Yes | Yes | Yes | Yes | Yes | Yes | Yes | Yes | Yes | Yes | Yes | Yes | Yes | Yes | Unclear | Yes |
| Were the study subjects and the setting described in detail? | Yes | Yes | Yes | Yes | Uncle  ar | Yes | Yes | Yes | Yes | Yes | Unclea  r | Yes | Yes | Yes | Yes | Yes | Yes | Yes | Yes | Yes |
| Was the exposure measured in a valid and reliable way? | Yes | Unclear | Unclear | Yes | Yes | Unclear | Yes | Yes | Yes | Yes | Yes | Yes | Yes | Yes | Yes | Unclea  r | Unclear | Unclear | Yes | Yes |
| Were objective, standard criteria used for measurement of the condition? | Yes | Yes | Yes | Yes | Yes | Yes | Uncle  ar | Yes | Yes | Yes | Yes | Yes | Yes | Yes | Yes | Yes | Yes | Yes | Yes | Yes |
| Were confounding factors identified? | Yes | Unclear | Yes | Yes | Yes | Unclear | Uncle  ar | Yes | Uncle  ar | Unclear | Unclea  r | Uncle  ar | Uncle  ar | No | Unclear | Unclea  r | Not applicabl e | Not applicable | Not applicabl e | Not applicable |
| Were strategies to deal with confounding factors stated? | Yes | Unclear | Unclear | Yes | Yes | No | Uncle  ar | Yes | Uncle  ar | Unclear | Unclea  r | Uncle  ar | No | No | No | Unclea  r | Not applicabl e | Not applicable | Not applicabl e | Not applicable |
| Were the outcomes measured in a valid and reliable way? | Yes | Unclear | Unclear | Yes | Uncle  ar | No | Yes | Yes | Yes | Yes | Yes | Yes | Yes | Yes | Yes | Unclea  r | No | Yes | Yes | Yes |
| Was appropriate statistical analysis used? | Yes | Yes | Yes | Yes | Yes | Yes | Yes | Yes | Yes | Yes | Yes | Yes | Uncle  ar | Yes | Unclear | Unclea  r | Yes | Yes | Yes | Yes |
| % | 100 | 50 | 62,5 | 100 | 75 | 50 | 62,5 | 100 | 75 | 75 | 62,5 | 75 | 62,5 | 75 | 62,5 | 37,5 | 50 | 62,5 | 62,5 | 75 |
